# Navigating Middle Lobe Syndrome: Exploring the Impact of Mycobacterium avium

**DOI:** 10.7759/cureus.67663

**Published:** 2024-08-24

**Authors:** Roberto Sanchez, Krystal Simono, Diana Duran, Cristal Liriano, Sergio Martinez

**Affiliations:** 1 School of Medicine, Universidad Iberoamericana, Santo Domingo, DOM; 2 School of Medicine, Pontificia Universidad Catolica Madre y Maestra, Santiago, DOM; 3 Pulmonology, Northwell Health, New York, USA

**Keywords:** case report, antibiotics, lung atelectasis, mycobacterium avium complex (mac), middle lobe syndrome

## Abstract

Middle lobe syndrome (MLS) is characterized by recurrent or chronic collapse (atelectasis) of the middle lobe of the right lung. Despite its clinical significance, MLS often goes unnoticed in medical practice. It manifests with obstructive symptoms, either due to external compression or internal causes, commonly stemming from infectious agents such as Mycobacterium avium complex (MAC) or occasionally from tumors. We present a unique case of MLS induced by MAC in an immunocompetent 74-year-old female patient with a history of bronchiectasis. Imaging revealed typical findings associated with MLS. Additional testing confirmed the diagnosis, and the patient was successfully treated. This case presents the opportunity to recognize and correctly treat cases of MLS with infectious etiology.

## Introduction

Middle lobe syndrome (MLS) is currently defined as the collapse of the middle lobe of the right lung and, occasionally, the lingula of the left lung [1]. This term was first used in 1948 when Graham et al. [2] reported the cases of 12 patients who presented with bronchial impingement by enlarged lymph nodes causing middle lobe atelectasis of nontuberculous origin. MLS is more common in females, although men and children are also affected. Most patients with MLS present with cough, chest pain, dyspnea, fever, chills, and audible wheezing [2]. However, some are asymptomatic and are diagnosed on routine chest X-ray (CRX).

It has been suggested that the anatomy of the right middle lobe bronchus, a long and small caliber structure surrounded by lymph nodes on both flanks, contributes to the pathologic process of MLS [3]. This is particularly relevant when the nodes are compromised by pathological processes, such as inflammation and poor lymphatic drainage, causing impingement on the lumen of the bronchus [1,3]. Pathophysiologically, two forms of MLS have been described: obstructive and nonobstructive. The obstructive subtype is caused by compression of the right middle lobe bronchus and can be attributed to both benign or malignant neoplasms, granulomatous infections, adenopathy, and aspiration of foreign objects (more often seen in the pediatric population), among other causes. In contrast, the nonobstructive type of MLS has no evidence of bronchial obstruction and can be a consequence of inflammation and edema in conditions such as asthma, cystic fibrosis, bronchitis, and recurrent bouts of pneumonia.

The diagnosis of MLS is made through CXR, in which the lateral view is preferred due to the abnormalities being more apparent. However, CXR may fail to reveal details that are crucial to establishing the diagnosis and treatment of MLS [2]. In addition, bronchography and flexible bronchoscopy are useful in excluding malignancy and collecting specimens for the diagnosis of infectious diseases [2]. The treatment of MLS for both the nonobstructive and the obstructive subtypes is directed toward treating the underlying cause. Conservative treatment consists of bronchodilators, mucolytics, and antibiotics. However, the effectiveness of antibiotic treatment on MLS warrants further evidence to be proven as an effective treatment [2,3]. For patients with evidence of malignancy or resistance to conservative management, middle lobe lobectomy can be offered [1].

## Case presentation

A 74-year-old female presented to the clinic with a constant productive cough with brownish-green sputum and chest congestion for the past three months. She denied travel history, close contacts, and smoking history. On physical examination, the patient had bilateral basilar rhonchi and wheezing. Blood pressure was 138/68 mm Hg, temperature was 97.8 F, BMI was 29.89 kg/m^2^, and oxygen saturation was 95% on room air.

**Figure 1:**
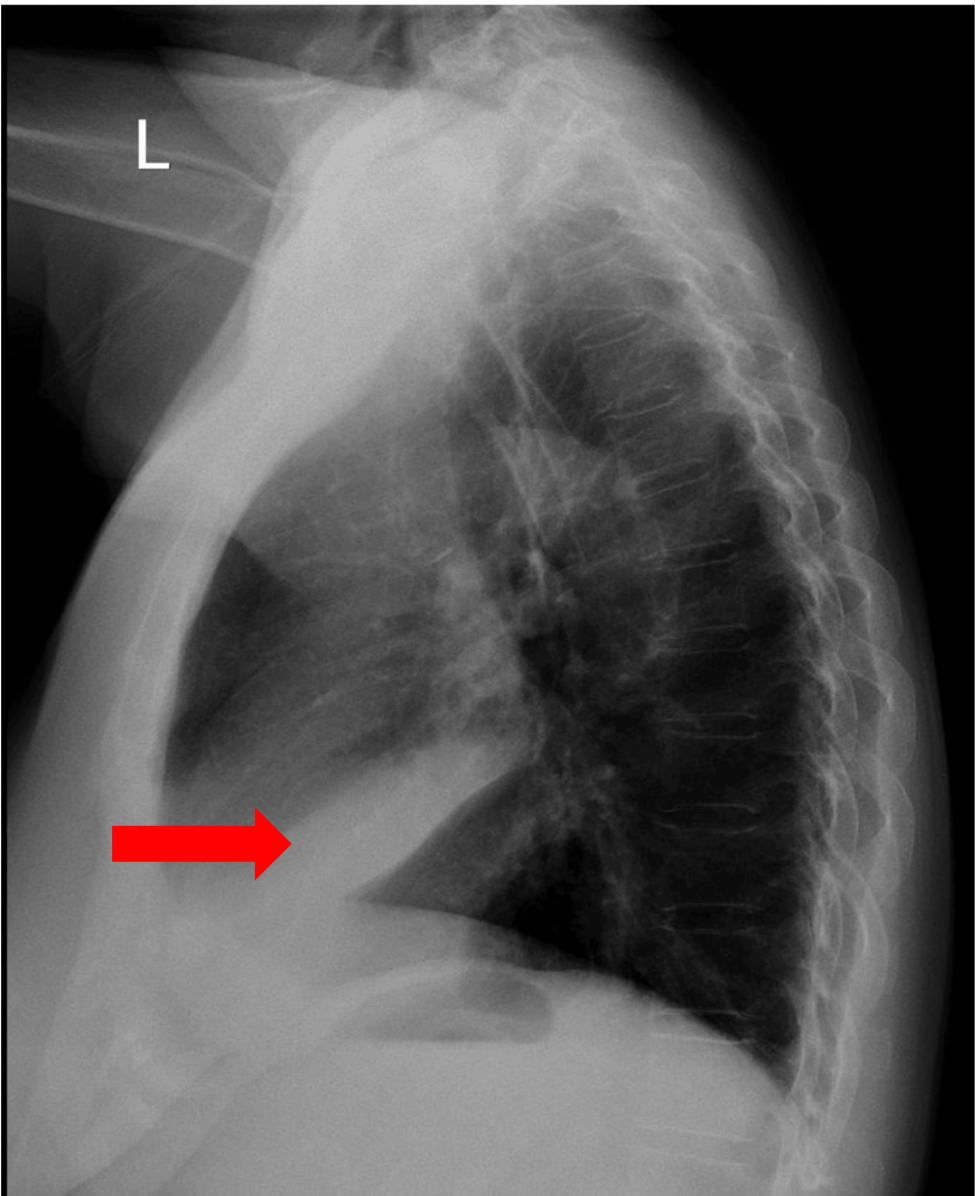
Lateral view of the chest X-ray showing consolidative opacity in the right middle lobe (red arrow), suggestive of infiltrates or atelectasis.

**Figure 2:**
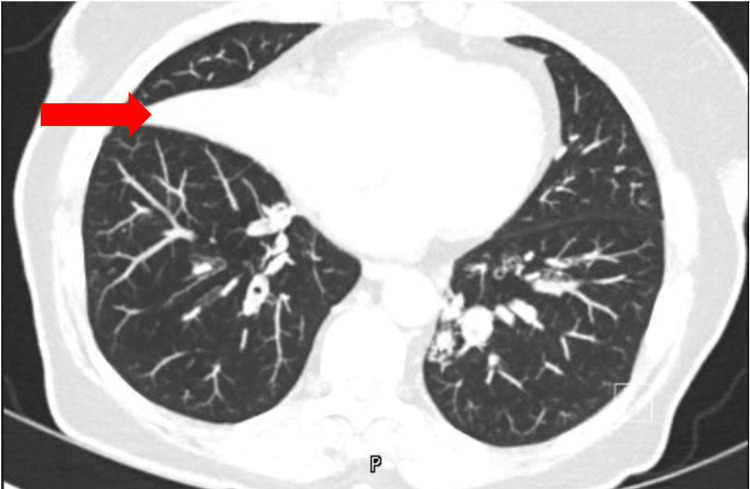
Axial plane of chest CT showing complete atelectasis of the right middle lobe and bronchiectasis with severe mucoid impaction in the left lower lobe.

Following the initial assessment with CXR (Figure 1), a decision was made to proceed with a chest CT scan (Figure 2) for further detailed evaluation.

The differential diagnosis included acute bronchiectasis exacerbation, chronic obstructive pulmonary disease exacerbation, and *Mycobacterium avium* intracellulare (MAI) complex infection. Therefore, she was initiated on fluticasone/umeclidinium/vilanterol 200 mcg-62.5 mcg-25 mcg (Trelegy) powder for inhalation once a day, a course of prednisone 20 mg once a day for seven days, and sputum cultures.

After three weeks of follow-up consultation, she was seen at the office, and she persistently complained of a productive cough, wheezing, and shortness of breath, although she stated that she was feeling better than the last visit. Until results for MAI were received, she was empirically treated with levofloxacin 250 mg two tablets a day, orally once a day for 10 days, a tapering of prednisone starting with 40 mg a day orally with tapering 10 mg every three days, and promethazine 6.25-15mg/5mL, 5mL as needed, orally for 10 days.

Results of MALDI-TOF (matrix-assisted laser desorption ionization - time of flight) showed positivity for isolated *Mycobacterium avium*. For this reason, she was initiated on azithromycin 500mg three times a week (TIW), rifampin 600 mg TIW, and ethambutol 25 mg/kg TIW for 12 months, along with Trelegy once a day. Additionally, she was treated with high-frequency chest wall oscillation (the vest) to improve mucus clearing, as well as pulmonary rehabilitation.

She was seen after a month of initiating the treatment for MAI, and she stated that she was feeling better than last time. She denies wheezing, shortness of breath, and cough.

## Discussion

As previously mentioned, both obstructive and nonobstructive etiologies can lead to the development of the MLS. Evidence shows that infections have been identified as a cause of MLS due to the predisposition of the middle lobe’s anatomical structure and the absence of collateral ventilation [4]. The middle lobe has a long, narrow diameter bronchus at an acute angle, which predisposes to infections due to decreased mucus clearance. In addition, the middle lobe encloses deep fissures that act as barriers to collateral ventilation. This isolation decreases the chances of reinflation after atelectasis has taken place [5].

Rashid et al. [4] reported that MLS has been related to microorganisms that include *Aspergillus *species, *Histoplasma* species, *Blastomyces* species, *Streptococcus pneumonia*, *Staphylococcus aureus*, and *Mycobacterium* species, among others. In the case discussed here, the microorganism detected corresponds to *Mycobacterium avium*. Pulmonary disease resulting from *Mycobacterium avium* complex (MAC) infection in immunocompetent individuals can be classified into primary and secondary forms. The first one mentioned occurs in immunocompetent nonsmoking individuals where the population commonly affected corresponds to elderly women (60-80 years old), which has been referred to as Lady Windermere syndrome. This condition is commonly seen in the right middle lobe due to bronchiectasis and chronic atelectasis where MAC plays a role [4,6,7]. In contrast, the secondary form occurs in people with underlying lung disease [4].

The initial diagnostic test for diagnosing MLS typically involves a posteroanterior (PA) and lateral CRX [5]. Abnormalities in CRXs are often more conspicuous in the lateral view [2]. One notable discovery is the triangular region of increased density, suggesting a decrease in volume due to the collapse of the right middle lobe. Detecting middle lobe collapse is often challenging on the posteroanterior projection, as the classic silhouette sign may be obscured by a right middle lobe infiltrate, especially in anterior-posterior (AP) or PA projections, given the relatively thin and oblique nature of the middle lobe [2].

High-resolution CT scans provide comprehensive views of MLS, facilitating the detection of bronchial abnormalities, parenchymal complications, and lymph node enlargement. Flexible bronchoscopy is vital for evaluating bronchial patency, identifying tumors, and procuring diagnostic samples. Histological analysis frequently uncovers bronchiectasis and foreign body reactions, while rarer discoveries encompass thrombi, arteriovenous malformations, and diverse inflammations [2].

The underlying etiology of MLS syndrome is the main focus of treatment. In cases where an infection is the cause as exposed in our case, antibiotics are frequently administered [4,8]. For MAC infections, the treatment consists of an antibiotic combination regimen of macrolide-ethambutol-rifampicin that should be given three times per week for 12 months [9]. Other treatment options for MLS include bronchodilators and mucolytics to improve breathing and postural drainage [10]. Another therapeutic approach is surgical excision of the middle lobe; however, this should only be used in persistent cases of MLS that are not improving with previous treatment [4].

According to several studies, MLS tends to have a favorable prognosis with proper treatment but can vary depending on the severity of the disease and the presence of complications. In a case presented by Carrillo-Muñoz et al., the patient improved significantly after starting treatment [11]. However, it is necessary to implement surgical measures in more serious situations or in situations in which the syndrome is resistant to medications, as was the case presented by Seitz et al. They concluded that thoracoscopic resection of the middle lobe seems safe and promising, comparable to other endoscopic procedures. A minimally invasive approach is preferred, when possible, after a thorough preoperative evaluation [12].

## Conclusions

MLS has a wide array of pathophysiological causes that most often result in the collapse of the middle lobe of the right lung. MLS is diagnosed through CXR; however, other diagnostic tests such as bronchoscopy and CT scan may be used to aid in the diagnosis as well. The treatment is directed toward dealing with the underlying cause, which may warrant antibiotic use, bronchodilators, and mucolytics. In cases of severe disease, surgery may be needed. It is imperative to understand and recognize MLS to provide patients with timely diagnosis and treatment, which potentially can lead to better patient care and outcomes.
